# Identification of the molecular mechanism of insulin-like growth factor-1 (IGF-1): a promising therapeutic target for neurodegenerative diseases associated with metabolic syndrome

**DOI:** 10.1186/s13578-023-00966-z

**Published:** 2023-01-23

**Authors:** Archana Arjunan, Dhiraj Kumar Sah, Minna Woo, Juhyun Song

**Affiliations:** 1 Department of Anatomy, Chonnam National University Medical School, Hwasun, Jeollanam-Do 58128 Republic of Korea; 2 Department of Biochemistry, Chonnam National University Medical School, Hwasun, 58128 Republic of Korea; 3 BioMedical Sciences Graduate Program (BMSGP), Chonnam National University, 264 Seoyangro, Hwasun, 58128 Republic of Korea; 4 Division of Endocrinology and Metabolism, University Health Network and and Banting and Best Diabetes Centre, University of Toronto, Toronto, ON Canada

**Keywords:** Alzheimer’s disease (AD), Insulin-like growth factor-1 (IGF-1), Metabolic syndrome (MetS), Neurodegeneration, Neuroprotection

## Abstract

Neurodegenerative disorders are accompanied by neuronal degeneration and glial dysfunction, resulting in cognitive, psychomotor, and behavioral impairment. Multiple factors including genetic, environmental, metabolic, and oxidant overload contribute to disease progression. Recent evidences suggest that metabolic syndrome is linked to various neurodegenerative diseases. Metabolic syndrome (MetS) is known to be accompanied by symptoms such as hyperglycemia, abdominal obesity, hypertriglyceridemia, and hypertension. Despite advances in knowledge about the pathogenesis of neurodegenerative disorders, effective treatments to combat neurodegenerative disorders caused by MetS have not been developed to date. Insulin growth factor-1 (IGF-1) deficiency has been associated with MetS-related pathologies both in-vivo and in-vitro. IGF-1 is essential for embryonic and adult neurogenesis, neuronal plasticity, neurotropism, angiogenesis, metabolic function, and protein clearance in the brain. Here, we review the evidence for the potential therapeutic effects of IGF-1 in the neurodegeneration related to metabolic syndrome. We elucidate how IGF-1 may be involved in molecular signaling defects that occurs in MetS-related neurodegenerative disorders and highlight the importance of IGF-1 as a potential therapeutic target in MetS-related neurological diseases.

## Introduction

Metabolic syndrome (MetS) is a collection of metabolic abnormalities, including hypertension, central obesity, and atherogenic dyslipidemia [1]. MetS significantly increases the risk of type 2 diabetes mellitus (T2DM) and cardiovascular disease [2]. Additionally, emerging evidences have shown that MetS can affect the central nervous system (CNS) diseases through various mechanisms [3]. Several studies suggest that MetS is associated with various neurodegenerative disorders, including Alzheimer’s disease (AD), Huntington’s disease (HD), and Parkinson’s disease (PD) [4–8].

Synaptic and glial dysfunction with aberrant networks between these cells is a hallmarks of neurodegenerative diseases (NDDs) [9]. Many NDDs can be classified as pyramidal and extrapyramidal, with motor and behavioral or cognitive impairments being the most common clinical manifestations [10]. Various molecular and cellular pathologies are associated with these NDDs, including oxidative stress, mitochondrial dysfunction, calcium (Ca^2+^) influx, glutamate toxicity, proteolytic stress, protein aggregation, neuroinflammation, and neuronal death [11, 12]. Over the past two decades, there has been a significant increase in evidence demonstrating the potent neuroprotective effects of neurotrophic factors (NTFs) on NDDs [13]. NTFs are crucial for CNS development and play vital roles in neurogenesis, neuronal cell migration, and CNS cell survival [14]. Recent research has focused on NTFs to understand their role in the etiology and as potential therapy for various neurological diseases. One of the major NTFs is insulin-like growth factor-1 (IGF-1), a peptide hormone (7649 Da and 70 amino acids) that belongs to the insulin-like hormone superfamily that include insulin, IGF-1, and IGF-2 [15].

The molecular signalling of IGF is highly evolutionarily conserved. IGF1 can act through autocrine, paracrine and endocrine mechanisms to regulate cellular growth, differentiation and proliferation [16]. The IGF system consists of six IGF binding proteins (IGFBPs) and two growth factors (IGF-1 and IGF-2) along with their cognate insulin growth factor receptors (IGF-1R, IGF-2R) [15]. The majority, up to 99% of IGF-1 binds to circulating IGFBP-1[17]. In the brain (hippocampus, cortex, olfactory lobes, cerebellum, and amygdala), IGF-1 binds to IGFBP-2, -4, and -5b [18]. In adults, IGF-1 is produced primarily in the liver and to a lesser extent in the hippocampus, cerebellum, and subventricular zone-olfactory bulb (SVZ-OB) under stimulation of growth hormone (GH) [19]. GH regulates neurogenesis and neuronal plasticity [20]. IGF-1 exerts its actions by binding and activating its membrane receptors, which are receptor tyrosine kinases [16]. After IGF-1 binds to its ligand, a series of phosphorylation events leading to activation of insulin receptor substrates, mitogen-activated protein kinase (MAPK), and phosphoinositide 3-kinase/protein kinase B (PI3K-Akt) lead to various intracellular processes [21]. A recent study has focused on the role and therapeutic potential of IGF-1 in the CNS to improve brain function and complex mechanisms of the CNS in MetS-induced neurodegeneration [22]. Herein, we focus on the potential therapeutic effects of IGF-1 in NDD associated with MetS and the molecular mechanisms underlying its pharmacological effects.

## IGF-1 in the CNS

**Fig. 1:**
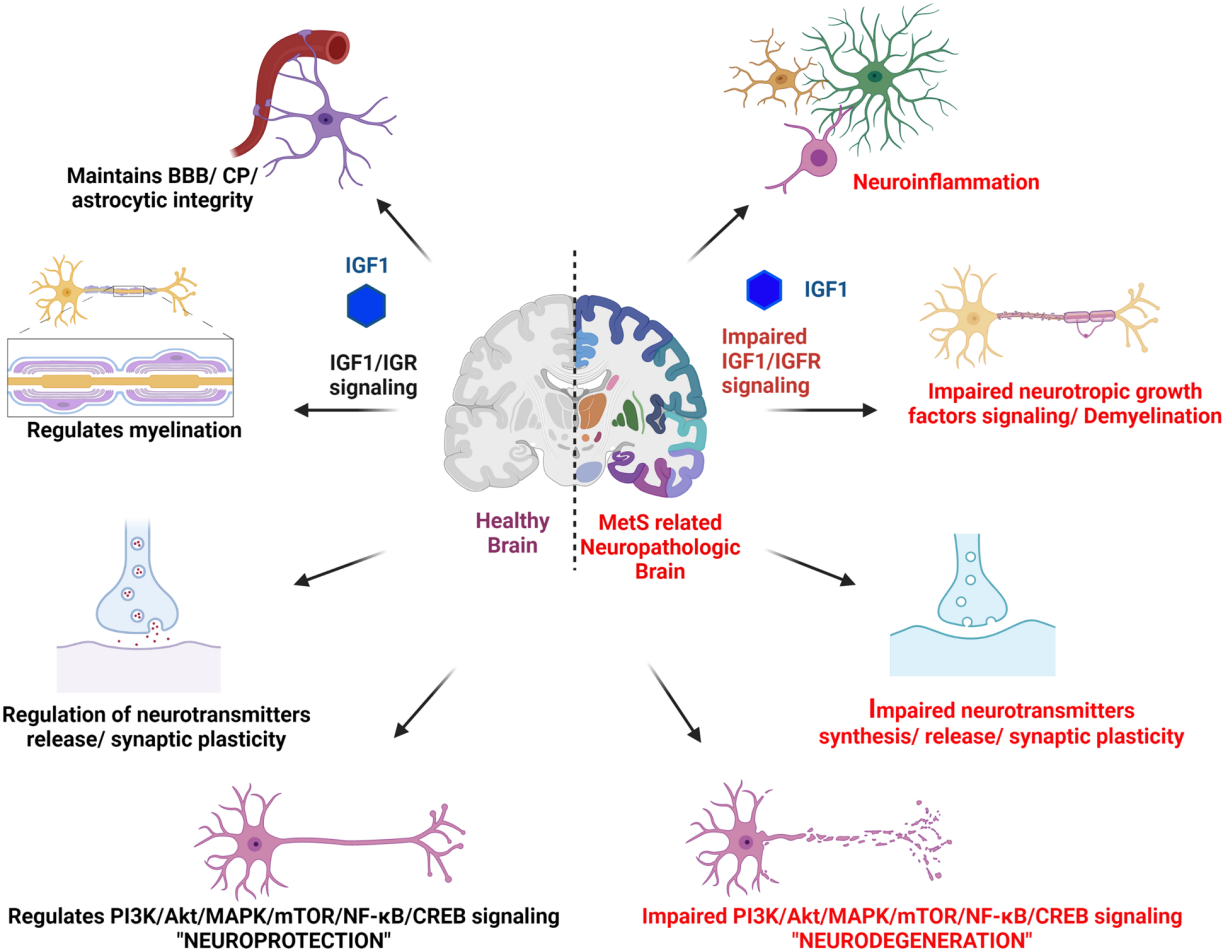
Schematic image on physiological and pathological action of IGF-1. IGF-1 in the healthy brain maintains the cerebrovascular microenvironment and BBB/CP integrity, regulates inflammation in microglia, and facilitates synaptic communication and cognition by acting on ionic channels and neurotransmitters. In Metabolic syndrome (MetS) related brain, both systemic and local deficiency of IGF-1 shows the altered cerebrovascular microenvironment/disturbed BBB/CP integrity, increased the deposition of α- Synuclein/Tau/Aβ/HTT proteins cause impaired neuroinflammatory action, neurotransmitter release, synaptic plasticity, and cognition which may lead to neurodegenerative diseases (AD/PD/HD)

IGF-1 can cross the blood brain barrier (BBB) and enter CSF, and perform a number of important functions of the CNS, including neurogenesis and neuroprotection, through autocrine/paracrine or endocrine effects. It affects metabolic regulation in the CNS, promotion of other nerve growth factors, clearance of aggregate proteins, and angiogenesis [23–25] (Fig. 1). High levels of IGF-1 are found in the CNS during early stages of organogenesis, which promotes brain derived growth factor (BDNF) and other neutrotropic factors that play important roles during brain development [26, 27]. Another study demonstrated that IGF-1 administration increased overall BDNF and decreased expression of interleukin (IL)-1β, TNF-α, nitric oxide synthase (iNOS), and glial fibrillary acidic protein (GFAP) in the whole brain [28]. IGF-1/IGF1R signaling has also been associated with Schwann cell (SC) survival, migration, proliferation, and myelination [29, 30]. In-vitro experiments with glial cells, oligodendrocytes, brain explants, and adult stem cells have revealed that IGF-1 promotes myelination, differentiation and mitogenesis [31]. Furthermore, IGF-1 can promote oligodendroglial cells to survive by inhibiting caspase-3 [31]. IGF-1/IGF-1R knockout mice showed decreased brain size, loss of myelination, and cognitive decline, whereas overexpression of IGF-1 resulted in increased brain size and myelination [32]. Moreover, IGF-1 regulates neural stem cell proliferation by promoting replicative lifespan and shortening all cell cycle lengths, particularly the G1/S transition [33]. Numerous clinical studies have demonstrated that IGF-1/IGF-1R mutations are associated with mental retardation and microcephaly [34, 35] (Table 2). Lichtenwalner et al., reported that altered levels of IGF-1 negatively affect neurogenesis and synaptic plasticity, particularly in the hippocampus [36]. In an in-vivo models, IGF-1 influences adult dentate gyrus development by increasing the number of granule neurons and thus increasing the dentate granule cell layer [37, 38]. IGF-1-RIT1-Akt-Sox2 pathway plays a key role in IGF-1-induced neurogenesis, cellular proliferation, and gene expression in hippocampus neurons [39]. IGF-1 can also influence neuronal excitability and glutamate system in brain [40]. In various in-vivo and in-vitro studies, exogenous administration of IGF-1 mediated has been shown to increase glucose utilization, release acetylcholine from neurons, activate N-methyl-D-aspartate receptor (NMDA), protect the cerebromicrovascular environment, and maintenance of synaptic structure and function [36, 41–45]. IGF-1 can also interact with NMDA receptors to promote synaptic function and facilitate PI3K/glutamatergic transmission in the hippocampus [46–49]. Kelsch et al. showed that during hippocampal maturation, K^+^/Cl^−^ outward transport is mediated by IGF-1/PI3K pathway [50]. Furthermore, IGF-1 increased presynaptic facilitation by activating p38/MAPK to modulate K^+^ channel activity [51, 52]. However, IGF-1R is highly expressed in cerebral plexus (CP), hypothalamus, thalamus, amygdala, and hippocampus/parahippocampal gyrus. Given that these regions are critically linked to cognition, it is compelling that IGF-1 and IGF-1R deficiencies lead to cognitive impairment [46, 53]. The transcriptional regulator CREB (cAMP response element binding protein) is a critical regulator of axonal growth and neuronal plasticity that is important for neuroprotection and cognition preservation [54]. In many cell types, IGF-1 enhances CREB phosphorylation and controls CRE-containing genes, such as c-Fos and B-Cell Leukemia/Lymphoma 2 (Bcl-2) [55]. Neuronal survival is also linked to the MAPK-CREB signaling pathway. By phosphorylating Bad and CREB, activated ribosomal protein S6 kinase beta (RSKs) can inhibit apoptosis [56, 57]. Also, IGF-1 can suppress various proapoptotic signals through regulation of multiple downstream targets [55]. As such, IGF-1 is an essential factor in maintaining neuronal homeostasis, and identifying the role of IGF-1 in the brain is important for finding clues to effective treatment targets for NDDs (Fig. 1).

## Therapeutic applications of IGF-1 in neurological diseases

Several recent studies highlight the pleiotropic actions of IGF-1 in neurons [29, 39, 42]. Tables 1 , 2 , and 3 describe the consequences of IGF-1 deficiency and the therapeutic effects of IGF-1 in experimental and clinical studies of neurological diseases. Our focus in this section is the effect of IGF-1 on neurodegenerative diseases, specifically AD and PD.

**Table 1:** IGF-1-deficient-induced neurological disease

No	Model	Findings	References
1	LID Mice	IGF-1 deficient cause neuro-glio-vascular unit damage	[188]
2	IGF1R (VE-Cadherin-Cre^ERT2^/Igf1r^f/f^)	IGF-1 is critical for cerebromicrovascular endothelial health and maintenance of normal neurovascular coupling (NVC) responses	[189]
3	IGF1R (GFAP-Cre^ERT2^/Igf1r^f/f^)	IGF-1 promotes astrocyte health and maintains normal NVC, protecting cognitive health	[45]
4	AD clinical study	The IGF-1 level was increased in AD subjects’ serum but not in CSF	[190]
5	Aged LID mice	IGF-1 is essential for the regulation of mitochondrial function, redox status, and cognition, and IGF-1 deficiency with age may increase brain damage and cognitive deficits	[191]
6	igfr^f/f^ mice	Reduced IGF-1 increases the accumulation of extrasynaptic glutamate, which may contribute to neurodegeneration in disease states	[192]
7	IGFR (GFAP-Cre^TAM^/igfr^f/f^)	Reduction in IGFR expression with age is associated with a decrease in hippocampal-dependent learning and increased gliosis	[193]
8	MS clinical study	Low serum IGF-1 was associated with cognitive impairment and fatigue in MS	[194]
9	AD and vascular dementia (VaD) clinical study	Low serum IGF-1 was a risk marker for VaD	[195]
10	AD clinical study	Lower baseline serum IGF-1 was associated with a faster cognitive decline in AD over a 2-year period	[196]
11	IGF-1 deficient mice (Igf1^f/f^ + TBG-Cre-AAV8)	IGF-1 deficiency exerts deleterious effects on cerebral microcirculation, causes a decline in cortical and hippocampal capillarity, and exacerbates hypertension-induced cerebromicrovascular rarefaction	[42]
12	AD clinical study	Increased levels of circulating IGF-1 and IGFBP-3 cause differences in mean age and MMSE scores, and circulating levels of IGFBP-3 decrease the level of IGF-1	[197]
13	AD clinical study	Lower serum IGF-1 was associated with cognitive impairment and was involved in the pathogenesis of cognitive deficits in AD	[84]
14	Postnatal/adult global IGF-I knockout (KO) mice (Igf-I2/2)	IGF-1 regulates postnatal/adult hippocampal neurogenesis in a stage-dependent manner	[198]
15	IGF-1 deficiency (Igf1(f/f)-TBG-Cre-AAV8)	IGF-1 deficiency also impaired glutamate-mediated CBF responses, likely due to dysregulation of astrocytic expression of metabotropic glutamate receptors and impaired mediation of CBF responses by eicosanoid gliotransmitters	[169]
16	IGF-1 deficiency (Igf1(f/f) -TBG-Cre-AAV8)	IGF-1-deficient mice included exacerbated disruption of the BBB and neuroinflammation that were associated with impaired hippocampal cognitive function	[164]
17	Prenatal stress/Dawley/Adult male offspring IGF-1, 10–20 µg/h/i.c.v	IGF-1 administration decreased IGF-1 levels and IGF-1 phosphorylation with altered IRS-1 phosphorylation in the hippocampus and frontal cortex of prenatal stress-induced rats	[199]
18	AD clinical study	Reduced serum IGF-1 is associated with the development of AD dementia in patients with AD	[82]
19	Clinical study IGF-1R mutation	IGF-1R mutations lead to prenatal and postnatal growth retardation and microcephaly	[27]
20	AD clinical study	Patients with AD as well as other dementias had high levels of IGF-1 in serum but not in CSF	[200]
21	AD clinical study	Low serum levels of IGF-1 and IGFBP-3 in males with AD but not in females with AD	[201]
22	Viral-mediated Cre-lox P system to knockout the Igf1 gene animal model	Adult-onset IGF-1 deficiency alone is sufficient to induce a depressive phenotype in mice Individuals with low brain IGF-1 levels are at increased risk for depression, and these behavioral effects are not ameliorated by increased local IGF-1 production or transport	[202]
23	AD clinical study	Significant decreases in IRS-1 and IRS-2 levels were identified in AD neurons in association with increased levels of inactivated phosphor (Ser312) IRS-1 and phosphor(Ser616)IRS-1, where increased levels of these phosphoserine epitopes colocalized strongly with NFTs	[203]
24	APP (SW), Tg2576 mice	Impaired IGF-1/IRS-2 signaling prevents premature death and delays amyloid accumulation in a model of AD	[204]
25	IGF-1R^±^ mice/MPTP induction	IGF-1R^±^ mice have shown increased dopamine neuronal loss in MPTP-induced mice	[96]
26	AD clinical study	Patients with vascular dementia and AD had low IGF-1 that may cause carotid atherosclerosis	[205]
27	Clinical cohort study IGF-1 and IGF-1R mutation	IGF-1 and IGF-1R mutant children had intrauterine growth retardation and poor postnatal growth	[26]
28	Brain injury-induced Romney-Suffolk fetal sheep/IGF-1 (3 or 30 μg/i.c.v)	IGF-1 treatment reduced caspase-3 activation and increased glial proliferation in a dose-dependent manner	[31]

**Table 2:** Therapeutic applications of IGF-1 in neurological diseases

No	Model	Findings	References
1	In vivo*/*in vitro AD model	In vivo transduction with RAd-IGF1 blocked memory impairment	[206]
2	Brain-specific IGF-1 overexpression mice	IGF-1 treatment reduced depressive and anxiety-like behavior, improved motor coordination, motor learning, visuospatial, and working memory	[207]
3	C57BL/6 J mice/controlled cortical impact/IGF-1	Increased immature neuronal density and neurogenesis of the hippocampus	[208]
4	RIT1^−/−^ mice/IGF-1	IGF-1 facilitates hippocampal neurogenesis through the RIT1/Akt/Sox2 signaling pathway	[39]
5	Old male rats/IGF-1	IGF-1 increases hippocampal neurogenesis and memory accuracy in aged individuals	[209]
6	Old Sprague–Dawley female rats/IGF-1, 18 days ICV	IGF-1 treatment increased the branching of hippocampal astrocytes and reduced their number in the hippocampal striatum radiatum, and improved spatial memory accuracy in aging rats	[210]
7	Female Sprague–Dawley rats/MCAo/IGF-1	IGF-1 reduced infarct volume (39%) and BBB permeability and suppressed IL-6, IL-10, and TNF-α	[211]
8	SH-SY5Y cells/10 nM IGF-1	IGF-1-induced shedding of both APP and APLP1 depends on PI3K, while APLP2 shedding is independent of this signaling pathway	[212]
9	In vitro/PD/IGF-1 along with MPP^+^	IGF-1 increases cell viability and decreases cell apoptosis	[213]
10	SH-EP1 cell lines/IGF-1 MPP^+^ neurotoxicity	Inhibition of MPP^+^-induced apoptosis by activating JNK by PI3K/AKT/GSK3β pathway	[106]
11	PD (WT, A30P and A53 T mutant)/100 ng/mL	Rescue from α-synuclein toxicity and suppression of α-synuclein aggregation	[214]
12	MT-IGF mice	Inhibits β-cell apoptosis, insulin secretion, and hepatic glucose production	[114]
13	Rat/6-OHDA/IGF-1 transgenic neurospheres	Reduction in amphetamine-induced rotation and increased survival of human neural progenitor cells (hNPC) exert trophic effects on degenerate dopamine neurons in the PD model	[103]
14	Adult female Long-Evans rats/6-OHDA/MPTP/IGF-1	By activating PI3K/Akt signaling, IGF-1 improved motor behavior and reduced DA loss in SNc	[105]
15	APP/PS2 mice IGF-1 (50 g/kg dose, i.p.)	Reverses spatial learning and memory impairment and reduces total brain Aβ deposition	[24]
16	Male Wistar rats/6-OHDA/GPE (3 mg/kg, i.p.)	Increased motor movement and reduced dopamine neuronal loss in PD rats	[101]
17	Adult female Long-Evans rats/6-OHDA/MPTP/IGF-1	IGF-1 significantly reduced the loss of asymmetric movement of the forelimb, reduced SNc neuronal loss, and TH immunoreactivity in DA fibers and striatum	[102]
18	Wistar rats/LID mice IGF-1: 50 µg/kg/rat/day	Reduced the brain Aβ burden and upregulated the brain levels of Aβ carriers	[83]
19	In vitro/IGF-1 (0.5 mg/mL)/dopamine	Decrease in apoptosis was accompanied by an increase in Bcl-2 levels	[4]
20	APP (WT-APP and V642I-APP mutant)/IGF-1: 10 nM	IGF-1 protected cells from APP-induced apoptosis and suppressed the cleavage of procaspase-3	[5]
21	Male BN × F344 rats/IGF-1 (50 ng/0.5 μL/h, i.c.v)	IGF-1 administration restored neurogenesis via a three-fold increase in neuronal production	[36]
22	Sprague–Dawley/hx rats/carotid artery IGF-1 infusion; 1.25 mg/kg per day)	IGF-1 increases progenitor cell proliferation and selectively induces neurogenesis in the progeny of adult neural progenitor cells in the hippocampus	[37]
23	Male Wistar rats/6-OHDA/GPE	A single dose of GPE increased TH immunoreactivity and reduced TH immunoreactive neuronal cell death in SNc and striatum	[100]

### Alzheimer’s disease (AD)

AD is characterized by a progressive cognitive decline affecting around 25 million individuals worldwide [58], and causes difficulties in learning and memory, language, and executive motor function [59]. AD is generally thought to be caused by amyloid-beta (Aβ) accumulation and plaque formation in the brain, a pathology known as the “amyloid hypothesis” [60]. There are evidences that IGF-1 may prevent age-related cognitive decline [48]. Several studies have shown that low IGF-1 levels are associated with AD (Table 1).

**Fig. 2:**
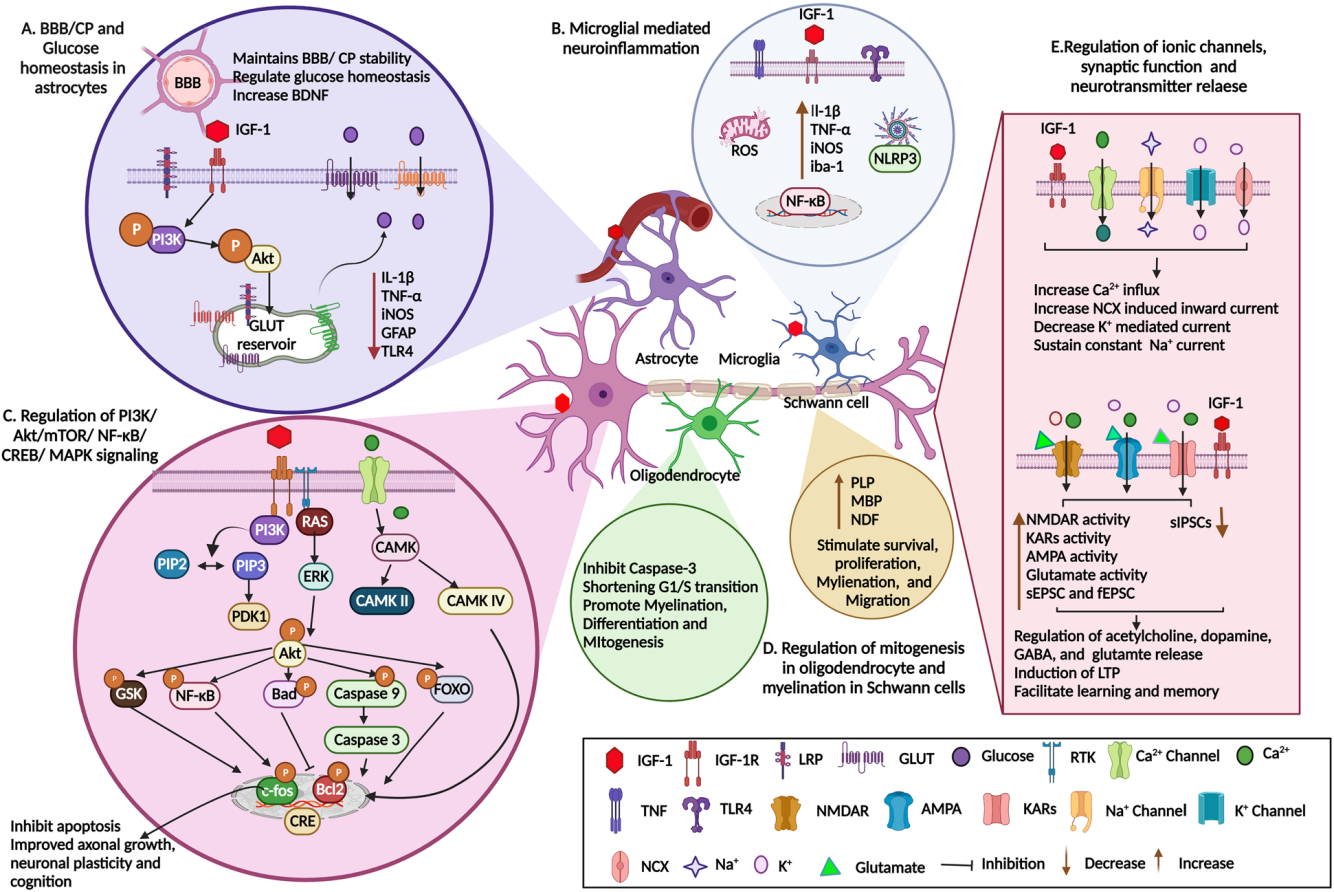
Schematic image on molecular actions of IGF-1 in CNS cells. **A** Blood–brain barrier (BBB)/choroid plexus (CP) and glucose homeostasis in astrocytes: IGF-1 binds to the astrocytic cell membrane's IGF-1 receptors, activates the PI3K/Akt pathway, and recruits the GLUT transporters, which then begins the uptake of glucose into the cell via GLUT transporters. **B** Neuroinflammation caused by microglia: When IGF-1 binds, it stimulates the polarization of the macrophages via TLR4 increasing the production of IL-1β, TNF-α, iNOS, and iba-1 while decreasing ROS and activating NF-κB/NLRP3 signaling. **C** PI3K/Akt/mTOR/NF-κB/CREB/MAPK signaling regulation in neurons: The PI3K/Akt signaling cascades are initiated when IGF- binds, phosphorylating the GSK, NF-κB, Bad, Caspase 9, and FOXO proteins. These additional phosphorylation result in the nuclear phosphorylation of c-fos and Bcl2, which prevents apoptosis, promotes axon development, and enhances neural plasticity. **D** Regulation of mitogenesis in oligodendrocytes and myelination in Schwann cells: In oligodendrocytes, IGF-1 inhibits the caspase-2 activity, shortening the G1/S cell cycle transition. In Schwann cells, IGF-1 facilitates myelination via increasing the myelinated proteins such as PLP, MBP, and NDF. **E** Regulation of ionic channels, synaptic function, and neurotransmitter release: IGF-1 regulates the Na^+^/Ca^2+^/K^+^ channels to increase the Ca^2+^ influx and maintain the Na^+^ concentration. In neurotransmitters, IGF-1 activates the NMDAR/KAR/AMPA receptors which regulate the acetylcholine, GABA, glutamate, and dopamine synthesis and release

Along with the canonical trophic role of IGF-1, it has also been shown to exert neuromodulatory effects through regulating neurotransmitter release (Fig. 2). Emerging research shows that glutaminergic neurotransmission through glutamate receptor NMDA plays a major role in learning and memory [61–63]. NMDA is also involved in the induction of long term potentiation (LTP) [63, 64] and can regulate synaptic plasticity [65]. Sonntag et al. reported that chronic administration of IGF-1 increases the density of NMDA receptors (NMDAR1, NMDAR2A, and R2B subunits) in the hippocampus, dentate gyrus and cortical areas, which are mainly involved in learning and memory [48]. Trejo et al. showed that IGF-1 restores cognitive function by attenuating the deposition of Aβ in an experimental model of AD [25].

**Table 3:** Therapeutic action of IGF-1 on MetS-related neurological diseases

No	Model	Findings	References
1	C57BL/6J mice/HFD/PEG-IGF-1	IGF-1 reduced anxiety-like depressive behavior and improved mitochondrial function via CREB/PGC-1α pathway	[183]
2	C57BL6/J mice/HFD LID mice (subcutaneous hIGF-1, 5 μg/kg/day)	IGF-1 increased sAPPα/sAPPβ ratio, increased peripheral Aβ clearance	[6]
3	Male C57BL/6J rats/HFD/HT22 cell line IGF-1, 1 mg/kg/4 weeks	IGF-1 enhanced cognition in HFD rats and inhibited inflammation and oxidative stress in the hippocampus through the activation of the PI3K/Akt/CREB pathway	[7]
4	Male Sprague–Dawley rats Adult Zucker diabetic fatty (ZDF) rats C57BL/6J mice hIGF-1, 20 µg/IP	IGF-1 increased CEBPβ overexpression, promoted neurite outgrowth, and mitochondrial respiration in diabetic animal models	[29]
5	Male Sprague–Dawley rats/STZ/IGF-1, 20 μg/subcutaneously	IGF-1 activates and upregulates AMPK to improve mitochondrial function, ATP synthesis, mtDNA copies, and ETS expression levels	[215]
6	R6/2 mice/IGF-1	Continuous peripheral administration of IGF-1 partially recovers plasma IGF-1 levels, inhibits HD-related glucose intolerance, protects from weight loss, and improves paw clasping scores	[8]
7	Male C57BL/6 N mice/STZ IGF1-AAV	IGF-1 improved motor function and reduced muscle atrophy and demyelination of the peripheral motor nerve fibers	[128]
8	STZ/MCAo model/IGF-1	Decreased lesion volume (CA1 and CA3 regions of the hippocampus and cortex) and reduced apoptosis	[216]
9	Sprague–Dawley rats/STZ/IGF-1	Prevented alteration of coenzymes (Q_9_ and Q_10_) and improved the antioxidant mechanism in diabetes-induced rat’s brain, liver, and kidney	[217]
10	IGF-1 Tg mice bred with IRS-1 null mutant (IRS-1^−/−^)	IGF-1 overexpression increased brain weight (43%) and promoted oligodendrocyte development and myelination	[130]
11	Male Sprague–Dawley rats/STZ/sc cell line-10 nM IGF-1	SCs are effectively protected against glucose-induced apoptosis by IGF-1	[218]

Furthermore, IGF-1 has been implicated in several ways to affect synaptic plasticity [66, 67]. IGF-1 may promote synaptic plasticity and transmission in minutes or persist for several hours to increase neuronal differentiation and survival (Fig. 2). Several types of neurons become more excitable in response to IGF-1 [68]. Studies have shown that the systemic administration of IGF-1 improves synaptic complexity and neurogenesis in the hippocampus [36, 69]. Moreover, IGF-1 in cultured hippocampal neurons increased the frequency of spontaneous excitatory postsynaptic currents (sEPSCs) for a short or long term, but had no effect on miniature excitatory postsynaptic currents (mEPSCs) or spontaneous inhibitory postsynaptic currents (sIPSCs). Indeed, the excitatory transmissions has been shown to be mediated by MAPK pathways [68]. Furthermore, IGF-1 inhibited synaptic transmission by increasing the frequency of sIPSCs in response to Aβ- reduction in sIPSC frequency [70]. This suggests that IGF-1 increased glutamate release at presynaptic sites or the functional excitability of synaptic contacts, but had no effect on non-NMDA or NMDA receptors.

In the CA1 region of the hippocampus, des (1–3)-IGF-1 increased the field excitatory postsynaptic potentials (fEPSPs), EPSCs, and α-amino-3-hydroxy-5-methyl-4-isoxazolepropionic acid receptor (AMPAR)-mediated postsynaptic exocytosis/endocytosis mechanism [71]. Des-IGF-1 affects glutamate receptor AMPAR binding protein (GRIP), N-ethylmaleimide-sensitive fusion protein (NSF), stargazin, and proteins that interact with C-kinase-1, which influence AMPA receptor anchoring, surface translocation, and synaptic targeting [72]. Furthermore, activation of the IGF-1R facilitates the AMPA synaptic mechanism by increasing intracellular calcium mobilization at the synapse [67]. These findings suggest that IGF-1 is important for regulating the AMPA receptors involved in LTP and cognition. IGF-1 modulates synaptic plasticity primarily by regulating ion channels (Ca^2+^-binding proteins), neurotransmitter secretion, and neuronal arborization. Also, IGF-1 phosphorylates and activates the α-1 subunit of the L-type Ca^2+^ channel through the PI3K pathway [73]. The Na^+^/Ca^2+^ exchanger (NCX) is a neuronal reciprocal Ca^2+^ transporter that promotes neuroprotection. This is mediated by IGF-1 by increasing the NCX-induced inward and decreasing the outward current [74]. Accordingly, systemic IGF-1 modulates the electrophysiological properties of target neurons. IGF-1 blocks transient A-type K^+^ currents and increases high-voltage-activated Ca^2+^ currents, while keeping low-voltage-activated Ca^2+^ and Na^+^ current constant [75].

Other glutamate receptors, including kainate receptors (KARs) and metabotropic glutamate receptors, can control long-term and short-term synaptic plasticity [76, 77]. KARs can be found on the gamma-aminobutyric acid (GABAergic) and glutamatergic presynaptic terminals [78]. IGF-1 increases the potency of kainate-dependent currents in cerebellar granule neurons and modulates Ca^2+^, Cl^−^, and K^+^ channels by PI3K-dependent pathway, but not MAPK dependent pathway [73, 79]. Although IGF-1 can stimulate neurogenesis and promote cognition in short-term, some studies have demonstrated that chronic administration of IGF-1 causes side effects such as accelerated aging, cancer development, and decreased lifespan [80, 81]. Thus, fine tuning these potential hormonal effects remains an important challenge to addressed.

Researchers have reported that reduced serum IGF-1 levels are associated with AD and decreased brain volume in clinical studies [82]. A study by Carro et al. showed that systemic administration of IGF-1 to mice deficient in hepatic IGF-1 resulted in increased serum IGF-1 levels, decreased the Aβ bodies in brain, and increased uptake by Aβ bodies in CSF facilitated by transthyretin and albumin [83]. Kimoto et al. also reported that reduced IGF-1 in serum is associated with cognitive deficits in subjects with AD [84]. IGF-1 can prevent AD development by altering several signaling proteins including rat sarcoma virus (Ras), forkhead box O (FoxO), and MAPK and their pleiotropic actions [85]. The IGF-1R belongs to the tyrosine receptor kinase family that controls many downstream targets, notably MAPK, Akt, Ras, PI3K, and the binding proteins growth factor receptor-bound protein 2 (Grb2) and Shc (Src homology 2 domain containing) transforming protein 1) [86]. PI3K/Akt is a well-known cascade induced by stimulation of IGF-1R [87]. Activated PI3K phosphorylates PIP2 to PIP3, which triggers the phosphorylation of P3-dependent kinase-1/2 (PDK-1/2) at Thr308 and Ser473 residues, resulting in Akt to be recruited to the plasma membrane (Fig. 2). The activated Akt can in turn phosphorylate various target proteins involved in survival and differentiation pathways, including BCL2 associated agonist of cell death (Bad), GSk3, nuclear factor-κB (NF-κB), FoxO1, FoxO3a, and FoxO4 [88]. IGF-1 suppressed NF-κB signalling by upregulating miR-219a-2-3p and inhibiting *YY1* gene expression, which is important for the activation of NF-κB signalling [89].

In AD, elevated levels of tumor necrosis factor-α (TNF-α) may play a significant role in exacerbating amyloidosis [90, 91], and IGF-1 attenuate amyloidosis by antagonizing TNF-α [83]. Recent researches have showed that altered CP function can exacerbate Aβ accumulation in the brain [73, 92], and numerous in-vitro studies have shown that IGF-1 can maintain tight junction stability in CP epithelial cells [92, 93]. On *in-vitro* study have also shown that IGF-1 maintains tight junction stability in CP epithelial cells [93]. Therefore, IGF-1 can modulate various ion channels and molecular signlaing pathways to attenuate inflammation and promote BBB-CP stability to prevent Aβ deposition and cognitive decline in AD.

### Parkinson’s disease (PD)

PD is the second common neurological diseases after AD, with a high incidence among adults in their 50s and 60s [94]. The neuropathological hallmarks of PD are a neuronal cell damage in substantia nigra of brain, leading to insufficient secretion of dopamine and accumulation of intracellular inclusions including α-synuclein aggregates [95]. Effective treatment for individuals with PD is challenging due to the lack of pharmacological options and adverse effects such as dyskinesia related to the use of levodopa.

In 1-methyl-4-phenyl-1,2,3,6-tetrahydropyridine (MPTP)-induced mouse model of PD, IGF-1R deficiency resulted in enhanced dopaminergic neuronal death [96]. Several clinical studies showed that low serum IGF-1 level was present in individuals with PD [97–99]. A cleaved form of IGF-1, glycine-proline-glutamate (GPE), prevents the death of tyrosine hydroxylase (TH) immunopositive neurons, and restores TH immunoreactivity in the substantia nigra compacta (SNc) and the striatum of a 6-hydroxydopamine (6-OHDA)-induced PD model [100].

Administration of cleaved IGF-1 (GPE-3 mg/kg, intraperitoneally [i.p]) also improved motor function and decreased dopaminergic neuronal loss in the 6-OHDA model [101]. Similarly, treatment with IGF-1 in 6-OHDA-induced PD model of ovariectomized rats resulted in increased motor function of the forelimbs, reduced loss of SNc neurons, and normal immunoreactivity of TH in the striatum and dopaminergic fibers [102]. In another study, IGF-1 significantly upregulated the survival of human neural progenitor cells in the 6-OHDA-induced PD model [103]. Alessandro et al. found that after depolarization, dopaminergic neurons secrete IGF-1, which can stimulate dopamine release in the ventral midbrain [104]. The neuroprotective effects of IGF-1 on PD are mediated by PI3K/Akt signaling rather than MAPK/ERK pathway [105]. Wang et al. reported that IGF-1 inhibits the activation of c-Jun N-terminal kinases (JNK) via the PI3K/AKT/GSK3β (Glycogen synthase kinase 3β) pathway, and 1-methyl-4-phenylpyridine ion (MPP^+^)-induced apoptosis [106]. Therefore, IGF-1 prevents the loss of dopaminergic neurons and improves motor function in PD model by upregulating the PI3K/AKT/GSK3/MAPK/ERK pathway.

## Metabolic syndrome and neuropathology

MetS is observed concurrently with several abnormalities including central obesity, hyperglycemia, hypertension, dyslipidemia, inflammation and thrombotic states [60]. The International Diabetes Federation criteria for MetS included a fasting blood glucose levels > 5.6 mmol/L (100 mg/dL); blood pressure > 130/85 mmHg; blood triglyceride levels > 1.7 mmol/L (150 mg/dL); HDL cholesterol levels < 1.0 mmol/L (40 mg/dL) for men and < 1.3 mmol/L (50 mg/dL) for women, and waist circumference > 94 cm (men) or > 80 cm (women) [107, 108]. Considering recent evidences, MetS is a major risk factor for type 2 diabetes (T2D) and cardiovascular disease, as well as an emerging major risk factor for NDDs.

Accumulating evidence supports that MetS plays a major role in the development of cognitive impairment [109]. MetS is also known to induce oxidative stress and inflammation, which can lead to cognitive decline by reducing the number and function of hippocampal neurons [109–112]. Furthermore, studies investigated the relationship between circulating IGF-1 concentrations and metabolic syndrome. This review focused on the neuroprotective effects of IGF-1 in MetS-related NDDs.

### Diabetes mellitus-related neurodegenerative disease

Diabetes mellitus is characterized by hyperglycemia due to complex pathogenic mechanisms involving widespread insulin resistance and impaired insulin production. Type 1 diabetes (T1D) is an autoimmune disease that causes damage to pancreatic β-cells. The most common type, type 2 diabetes (T2DM), is characterized by dysfunctional β cells and insulin resistance [113]. Scientific evidences has demonstrated a substantial association between diabetes (both T1D and T2D) and cognitive decline leading to dementia in animal models and humans [29, 49, 114, 115].

One study shows that 56% of AD dementia area associated with T2D [116]. In fact, the significance of the link between T2D and AD is now defined by the term “type 3 diabetes”, which describes a subset of diabetic patients who develop AD dementia [117–119]. In T2D, insulin resistance and altered IGF-1/IGF-1R signaling are associated with cognitive decline, Aβ production, tau hyperphosphorylation, proinflammatory marker’s expression, oxidative stress, and dyslipidemia [120, 121]. Rui-Hua et al. found that decreased serum IGF-1 levels were associated with T2D-associated cognitive decline in clinical trials [122]. Another study showed that subjects with mild cognitive impairment with T2D had a reduced serum IGF-1/IGFBP-3 molar ratio [123]. Aksu et al. showed that reduced IGF-1 induces anxiety-like behavior and reduced blood flow to the prefrontal cortex in streptozotocin (STZ)-induced diabetic rats [124]. In addition, Jing et al. showed that maternal hyperglycemia reduces the expression of IGF-1, resulting in delayed fetal dendrite development in STZ-induced rats [125].

Hyperglycemia is associated with a lack of neurotrophic signaling that can lead to mitochondrial dysfunction of SC [126]. Chronic hyperlgycemia can lead to vacuolization and atrophy or degeneration of myelinated nerve fibers [127]. Myelinated nerve fibers (Aδ-type afferent fibers) are susceptible to dysfunction when their conduction velocity changes [127]. Chu et al. reported that STZ-induced mice carrying an IGF-1 adeno-associated viral (AAV) vector showed reduced peripheral motor nerve fiber demyelination [128]. SC express IGF-1 receptor, and activation by IGF-1 stimulates myelination, attachment to axons, and migration [129]. Ping et al. found that in the cerebral cortex and brainstem, IGF-1 increased the expression of proteins essential for myelination, such as the proteolipid protein (PLP) and myelin basic protein (MBP)[130]. IGF-1 promotes Po induction, DMA synthesis, and DNA synthesis caused by neuro-differentiation factor isoforms in SC. These findings demonstrate that IGF can stimulate proliferation and differentiation in SCs [128].

IGF-1 forms the central core elements of astrocyte functions, such as the regulation of glucose uptake, glutamate transport, and protection against oxidative stress in the brain [44, 131, 132]. IGF-1R enters astrocytes by binding to astrocyte glucose transporter 1 (GLUT1) via the low-density lipoprotein receptor-related protein-1 (LRP1) and scaffolding protein GIPC PDZ domain containing family, member 1 (GIPC1) (Fig. 2). These results suggest that IGF-1R modulates brain glucose metabolism by inhibiting the activity of GLUT1 in astrocytes [44]. Another study demonstrated that IGF-1 increased hypoxia-inducible factor-1 (HIF-1) and GLUT3 protein expression to maintain glucose homeostasis in neurons through PI3K/Akt/mTOR-dependent pathway [133]. These results imply that astrocytes may be important sensors of peripheral hormonal changes that connect the cerebral microenvironment to neurons to respond to endocrine signals. Therefore, therapeutic targets for improving astrocytic function include enhancement of IGFR signalling and mitochondrial function and glucose transport, which can alleviate age-related pathologies such as AD (Fig. 2).

On the other hand, in diabetic mice, IGF-1 expression was significantly decreased and pain, neuroinflammation, and M1 microglial polarization were increased [134]. Microglia are highly dynamic and can adopt wide-ranging responses to their environment to govern CNS homeostasis [135]. In brain injury, the microglia response switches from a proinflammatory M1 to an anti-inflammatory/reparative M2 for recovery. If this process is not regulated, excessive reactive nitrogen species (RNS), ROS, and inflammatory cytokines secreted by M1 phenotype microglia can cause neuronal damage [135–137]. IGF-1 is mainly produced by microglia, which is elevated during the inflammatory process [138, 139]. IGF1 as a pleiotropic hormone, signals macrophages to help various tissues develop and maintain homeostasis [140]. Sun et al. reported that IGF1R stimulates M1 polarization through toll-like receptor (TLR4)/NF-κB pathway in intracerebral haemorrhage (ICH) induced mice [141]. Furthermore, IGF-1 activates the PI3K/Akt/FoxO1 pathway without affecting TLR2/4 expression in an in vitro hyperglycemic study [142]. Wolters et al. demonstrated that IGF-1 does not produce cytokine itself, and regulates TLRs responsible for inflammatory effects during metabolic complications. Another study reported that TLR4 mutant mice fed HFD showed neurovascular protection by improving astrocytic vascular recovery and cerebromicroenvironement [143]. Similarly, Maria et al. reported the anti-inflammatory action of IGF-1 in astrocytes by IGF-1 gene therapy. Additionally, exogenous treatment with IGF-1 reduced TLR4 expression and reduced NF-κB activation in lipopolysaccharide-induced inflammatory response of astrocytes [144]. As a result of TLR activation, downstream signaling pathways such as PI3K/Akt/mTOR and MAPK are induced, and promote cytokines production through activation of the NF-κB signaling pathway. These downstream targets are shared by the IGF1 receptor and TLRs [145]. Lee et al. demonstrated that IGF-1 exerts anti-inflammatory action by downregulating the TLR4 signaling in skeletal muscle [146]. These findings suggest direct pro/anti-inflammatory actions of IGF-1 which regulates neuroinflammation and is involved in neuroprotection by maintaining the cerebromicroenvirontment, increasing the capillary density and microglial activation in neuroinflammation by TLR4 signaling (Fig. 2). These findings imply that decreased IGF-1 levels are directly related to cognitive impairment, and neuroinflammation, and suggest that therapeutic restoration of IGF-1 levels may improve cognitive function.

### Obesity-related neuropathology

The prevalence of MetS has increased dramatically in the past decades, primarily due to significant lifestyle changes, including imbalance diet and physical inactivity [147]. According to recent estimates, around 2.1 billion people are overweight or obese [148]. Obesity has become a global epidemic with enormous medical, social, and economic burdens. Western diets are high in salt, processed carbohydrates and saturated fats, which negatively impact body mass and metabolism, including dyslipidemia, abdominal obesity, and T2D [149].

Obesity negatively affects CNS homeostasis and cognitive function [148, 150, 151]. The CNS and peripheral nervous system are fundamentally different in structure and function. And since both are prone to obesity-related dysfunction, this suggests a common pathway leading to the persistent disease progression through visceral fat. Also, a high body mass index (BMI) (> 30 kg/m^2^) has been recognized as one of the risk factors for PD [152, 153]. Obese individuals have fewer striatal dopamine receptors than non-obese individuals. Obesity has a deleterious influence on motor function and manual dexterity [154, 155].

Additionally, Bhat et al., reported that a high-fat/high-cholesterol diet can promote cognitive decline and brain dysfunction [156]. High fat diet (HFD)-induced obesity altered the circulating IGF cascade and increased circulatory level of total IGF-1, IGF-2, free IGF-1, and IGFBP3 in rodent and clinical trials [157, 158]. However, insulin/IGF signaling (IIS) may be critical in diet-induced AD-like pathology. Tau phosphorylation and GSK3 activation mainly result from impaired IIS signaling in the brain [159, 160]. Naryan et al*.* reported that downregulated IIS increased tau phosphorylation, promoted GSK activation, and decreased insulin receptor substrate-1 (IRS1), phospho-Akt, drebrin, and postsynaptic density (PSD95) resulting in cognitive impairment in the HFD model [156]. Based on these studies, obesity has been identified as one of the major causes for the development of neuropathology, and altered insulin/IGF signaling contributes to obesity-related AD.

### Cardiovascular disease-related neuropathology

Hypertension is defined as systolic blood pressure (SBP > 140 mmHg) or diastolic blood pressure (DBP > 90 mmHg), and is found in more than one billion people worldwide [161]. Hypertension plays a cardinal role in the progression of cerebromicrovascular injury and vascular cognitive impairment [42]. Some studies suggest that changes in cerebral microcirculation play a crucial role in age-related cognitive decline [162–164]. Furthermore, circulating IGF-1 has been shown to be a critical vasoprotective factor that declines with age, and its deficiency can accelerate vascular aging [165]. IGF-1 deficiency is also associated with an increased risk of early atherosclerosis and cerebrovascular disease [165]. Tarantini et al. reported that IGF-1 deficiency accelerates BBB damaged by hypertension, altered capillary morphology in cortical areas, and exacerbates neuroinflammation [42]. Additionally, HFD-fed GH/IGF-1 deficient animals showed glucose intolerance, increased body fat content, oxidative stress, activated inflammatory markers (TNF-α, ICAM-1), and endothelial dysfunction resulting in cerebrovascular damage [166].

Cerebromicrovascular rarefaction leads to decreased cerebral blood flow, which can lead to neurological dysfunction by lowering metabolic factors required for neural signalling [167]. Angelini et al. has been shown that decreased IGF-1 reduced acetylcholine release in the hippocampus, and ultimately led to cognitive decline in hypertensive subjects [43]. Sonntag et al. also showed that IGF-1 influences learning and memory function by regulating K^+^-induced acetylcholine release in the cortex and hippocampus [48]. Endothelium-derived nitric oxide (NO) is a key regulator of microvascular endothelial cell survival and a negative modulator of vascular endothelial growth factor (VEGF) signaling. IGF-1 deficiency impairs endothelial NO bioavailability through elevation of NO breakdown due to increased generation of reactive oxygen species (ROS) and downregulation of endothelial nitric oxide synthase (eNOS) [168, 169].

Other factors contributing to hypertension-related vascular dementia are aging and mitochondrial dysfunction. Reduced mitochondrial biogenesis, neuronal and astrocyte function, and increased ROS are important determinants of aging and neurodegeneration [170–173]. Mitochondria consume about 90% of cellular oxygen through cellular respiration, resulting in a constant stream of free radicals that, if mismanaged, cause long-term oxidative stress and damage [174–176]. IGF-1 reduces the pro-oxidant protein thioredoxin-interacting protein 1 and normalizes ROS levels (Fig. 2). Furthermore, IGF-1 can provide neuroprotection from oxidative danage by interacting with trophic factors secreted by astrocytes in conjunction with H_2_O_2_, such as stem cell factor (SCF) [132].

The IGF-1 pathway is a major determinant of aging. The rate of aging also depends on the amount of IGF-1 and the density of its receptors [177]. There is a considerable increase in neural MAPK phosphorylation with aging along with a decrease in Calcium/Calmodulin-Dependent Protein Kinase IIa (CaMKIIa) levels. Changes in the phosphorylation of synaptic kinases (CaMKII and MAPK) involved in the regulation of long-term potentiation may be related to IGF-1/IGF-1R signalling [158]. IGF-1 was recently identified as belonging to a new class of ion channel modulators with rapid response (Fig. 2). IGF-1 regulates N-type and L-type Ca^2+^ channels required for neuronal survival and release of neurotransmitters [178]. L-type Ca^2+^ channels (CaV1.2 and CaV1.3) regulate a wide range of neurological functions [179, 180]. IGF-1 can rapidly activate CaV1.3 by modulating IGF-1R, which phosphorylates and activates CaMKII (CaV1.3a and CaV1.3b at the C termini sites, resulting in inositol trisphosphate (IP3)-induced Ca^2+^ release [181]. CaV1.3 phosphorylation by IGF-1 at S1486 residue induces a left-shifted current–voltage that regulates CREB. Excitatory neurotransmitter-induced signaling pathways in the hippocampus are influenced by IGF-1-induced CREB/CaV1.3 signaling. IGF-1 increased Ca^2+^ influx through L-type Ca^2+^ channels and increased CaMK-IV activity, which reduced the expression of CCAAT enhancer-binding proteins (C/EBPβ) [182].

IGF-1 has been shown to improve mitochondrial function and transmembrane potential in a HFD-fed obese mouse model [183]. IGF-1 contributes considerably to vascular health and protects cells from vascular damage and neuropathological problems [169].

## Limitations and future perspectives

Although substantial research has been conducted on the IGF-1 signaling pathway in the past decades, the precise relationship between IGF-1 and cognition remains unclear. Although most studies using animal models have demonstrated neuroprotective effects, human studies have been less conclusive.

Despite its potential therapeutic significance outlined in this review, the long-term benefits of IGF-1 remain controversial. Various side effects have been reported with chronic IGF-1 therapy, including pain at the injection site and lipohypertrophy, headache, hypoglycemia, papilloedema, cataract, neoplasia, renal hypertrophy, and facial nerve palsy [80, 184]. Few studies have shown that overexpression of IGF-1 increases cancer risk through activation of the IRS/Akt/MAPK pathway [31, 80, 81, 120, 185]. Also, Ter Braak et al. mentioned that chronic administration of IGF-1 and its analogue promotes mammary tumor development in the p53R270H/+WAPCre mouse model [186]. Studies have shown that the IGF signaling pathway is not only involved in tumorigenesis, but also contributes to resistance to standard cancer therapies [187]. Whether these unwanted effects may outweigh the benefits in the long run remains an important area of further study. This review has primarily focused on research over the past few decades on the metabolic effects of NDDs. More rigorous studies taking a genetic approach are needed to evaluate the role of IGF-1 and its precise downstream mechanistic targets that provide neuroprotection.

## Conclusion

IGF-1 is a master regulator of protein, RNA and DNA synthesis and is involved in Ca^2+^ signaling that regulates synaptogenesis, neurite and glial (astrocytes, oligodendrocytes, schwann cells and microglia) proliferation and repair. Numerous studies have shown the neuroprotective effects of IGF-1. Thus, IGF-1 is a promising therapeutic option for the treatment of various neurological disorders through regulation of multiple neuroprotective signaling pathways, including Ras/Erk1/2, PI3K/MAPK/Akt/mTOR, Ca2+/CaMK II and IV, CREB, C/EBPβ, and GSK3B/NF-kB/NLRP3. It is also involved in regulating neuron and glial homeostasis through regulating ion channels, releasing neurotransmitters, and maintaining synaptic plasticity. Despite significant scientific advances supporting the restorative effects of IGF-1, the precise molecular pathways leading to its neuroprotective effects remain unclear and more studies are needed to accurately understand the role of IGF-1 in MetS-related neurological diseases.

## Data Availability

Not applicable.

## Abbreviations

AD: Alzheimer’s disease
Aβ: Amyloid beta
AMPK: AMP-activated protein kinase
AMPAR: α-Amino-3-hydroxy-5-methyl-4-isoxazolepropionic acid receptor
APP: Amyloid precursor protein
APLP1: Paralogues amyloid precursor-like protein 1
ATP: Adenosine triphosphate
AAV: Adeno-associated viral
Bad: BCL2 associated agonist of cell death
BBB: Blood–brain barrier
BCL-2: B-Cell leukemia/lymphoma 2
BDNF: Brain-derived growth factor
BMI: Body mass index
Ca^2+^: Calcium ion
CaMKII: Calcium/calmodulin-dependent protein kinase II
CBF: Cerebral blood flow
CNS: Central nervous system
CSF: Cerebrospinal fluid
CP: Choroid plexus
Cl^−^: Chloride ion
CREB: CAMP response element binding protein
C/EBPβ: CCAAT enhancer-binding proteins
DAYLs: Disability-adjusted life-years
DA: Dopamine
DCN: Dorsal column nuclei
ERK1/2: Extracellular regulated kinase 1/2
DM: Diabetes mellitus
eNOS: Endothelial nitric oxide synthase
fEPSPs: Field excitatory postsynaptic potentials
FoxO: Forkhead box O
GABA: Gamma-aminobutyric acid
GBD: Global burden of diseases
GCL: Granule cell layer
GFAP: Glial fibrillary acidic protein
GH: Growth hormone
GSK3: Glycogen synthase kinase
GRIP: Glutamate receptor AMPAR binding protein
GPE: Glycine-proline-glutamate
GLUT: Glucose transporter
GIPC: GIPC PDZ domain containing family: member 1
Grb2: Growth factor receptor-bound protein 2
HD: Huntington’s disease
HFD: High-fat diet
HIF-1: Hypoxia-inducible factor-1
hNPC: Human neural progenitor cells
ICH: Intracerebral haemorrhage
i.c.v: Intracerebroventricular
IGF-1: Insulin-like growth factor 1
IGF-1R/2R: Insulin-like growth factor 1 receptor
IGF-1/RTK: Insulin-like growth factor-1/receptor tyrosine kinase
IGFBP: IGF binding protein
IIS: Insulin/IGF signaling
i.p: Intraperitoneally
iNOS: Nitric oxide synthase
IP3: Inositol trisphosphate
IRS1: Insulin receptor substrate-1
IL: Interleukin
JNK: C-Jun N-terminal kinases
KARs: Kainate receptors
LID mice: Liver IGF-1 deficient mice
LID: Levodopa-induced dyskinesia
LRP1: Low-density lipoprotein receptor-related protein-1
LTP: Long-term potentiation
MCI: Mild cognitive impairment
MAPK: Mitogen-activated protein kinase
MBP: Myelin basic protein
mtDNA: Mitochondrial DNA
MT-IGF-1: Monocyte/macrophage-derived IGF-1
NCX: Na^+^/Ca^2+^exchanger
NDDs: Neurodegenerative diseases
NTF’s: Neurotrophic factor
NMDA: N-methyl-d-aspartate
MetS: Metabolic syndrome
MPTP: 1-Methyl-4-phenyl-1,2,3,6-tetrahydropyridine
MPP^+^: 1-Methyl-4-phenylpyridine ion
NF-κB: Nuclear factor-κB
mEPSCs: Miniature excitatory postsynaptic currents
NSF: N-ethylmaleimide-sensitive fusion protein
NO: Nitric oxide
NVC: Neurovascular coupling
K^+^: Potassium ion
PD: Parkinson’s disease
PDK-1: P3 dependent kinase-1
PI3K: Phosphatidylinositol 3-kinase
PLP: Proteolipid protein
PSD95: Post synaptic density 95
Ras: Rat sarcoma virus
RNS: Reactive nitrogen species
ROS: Reactive oxygen species
RSKs: Ribosomal protein S6 kinase beta
SC: Schwann cell
SCF: Stem cell factor
sEPSC: Spontaneous excitatory postsynaptic current
sIPSCs: Spontaneous inhibitory postsynaptic currents
SNc: Substantia nigra compacta
Shc: Src homology 2 domain containing-transforming protein 1
STZ: Streptozotocin
SVZ-OB: Subventricular zone-olfactory bulb
TH: Tyrosine hydroxylase
TNF-α: Tumor necrosis factor-α
TLR4: Toll-like receptor 4
T2DM: Type 2 diabetes mellitus
ZDF: Zucker diabetic fatty
6-OHDA: 6-Hydroxydopamine
VaD: Vascular dementia
